# The health consequences of economic crises

**DOI:** 10.3134/ehtj.09.004

**Published:** 2009-06-09

**Authors:** AG Robertson

This journal is dedicated to researching and publishing on emerging threats to human health. The subject areas vary from the threats to a small group, such as Iranian veterans exposed to sulphur mustard, through to threats on a regional or global basis. One of the key evolving threats is the current global economic crisis.

In October 2008, the world finally realised that the credit crisis, which arose from the United States sub-prime housing collapse, was the harbinger of the most significant international recession since the 1930s.^1^ Although the economic and political consequences of the recession are being studied in exhaustive detail, the potential health consequences have received much less attention.

The effects on health are likely to be manifold. At a global level, health care, already precarious in many developing countries, is likely to decline further as aid dries up and government expenditure falls, with millions more forced into poverty and malnutrition, particularly women and children.^2^ This is likely to be further exacerbated by the collapse of business and banking institutions as world trade declines, on a background of raised food and fuel costs.^3^

The consequences have been seen before. After the Asian crisis in 1997, women and girls generally suffered disproportionately as the industries that employed them were the first to be hit, and as spending on women's health care, including antenatal and maternity services, was cut.^4^ Excess deaths can also be expected, particularly in developing countries, as poorer patients defer medical treatment and health services deteriorate, with the greatest impact likely to be on the most vulnerable patients including children, the disabled, and the elderly.^5^ After the Mexican financial crisis in 1995, over 27,000 excess deaths were estimated to have occurred in these vulnerable groups.^6^ In more developed countries, mortality rates may actually fall as a result of reduced alcohol and tobacco use, and less road travel.^7^

Disease control is also expected to suffer during the current crisis. There is growing concern that funding for treatment, research, and global control of malaria, tuberculosis, and HIV/AIDS is being cut back, and that other currently underfunded programs for serious infectious diseases would not get the investment they require.^8^ Resources for disease surveillance and laboratory capacity are also often cut back at these times, which may seriously impact on the timely identification and mitigation of emerging epidemics and pandemics.^9^

Even in more developed countries, health care and services are expected to suffer. The effects will be varied, depending on the mix of public and private healthcare institutions in the system, but will have a common theme. The financial downturn is already making private health care less viable for an increasing percentage of people,^5^ particularly in those developed countries where private health care forms a significant sector of the overall healthcare system. This places increased pressure on publicly funded health systems also expected to suffer as state and national coffers dry up. During the 1998 Asian financial crisis, there was a significant shift of patients from private hospitals to public health facilities.^7^ Patient volumes, particularly for elective surgery, are already dropping in private hospital systems in the United States.^10^

Having a national public health system will not be a bulwark either. As unemployment rises, ill health and demand for healthcare services, including mental health and elective surgery, are also expected to rise.^5^, ^11^ As governments will be simultaneously trying to reduce expenditure from their shrinking budgets, by reducing health workforce numbers and healthcare costs during a time when demand continues to increase, the strains on healthcare services will continue to intensify.^3^

Finally, the political and social impacts of the financial crisis are just starting to be felt. As unemployment rises and people's livelihoods deteriorate, the effectiveness of respective governments in addressing the crisis is being questioned. Protests in both developed and developing countries, from Ireland to Senegal, are starting to occur. How these protests are addressed by various governments will determine whether they remain relatively peaceful or continue to escalate into increasing violence, with refugee groups from neighbouring countries often being the focus of that violence. In some instances, this may extend across borders with conflicts between neighbouring countries, which may also serve as a distraction from domestic problems. The economic crisis may also contribute to increased extremism and terrorism in countries that struggle economically, particularly where developed countries are perceived to be responsible.^12^ We should not be quick to forget the political, social, and subsequent military impacts of the Great Depression.^1^

The ultimate impact of the current crisis on health and health services globally will not be known for years to come, although we can probably safely assume that it will not be positive. As a journal dedicated to emerging health threats, we propose that urgent research and critical analysis are necessary to look into the impact that this economic crisis is having on health in both developed and developing countries, and into identifying solutions than may ameliorate the most detrimental effects of this potential healthcare disaster.
